# A chromosome-level genome assembly of *Drosophila madeirensis*, a fruit fly species endemic to the island of Madeira

**DOI:** 10.1093/g3journal/jkae167

**Published:** 2024-07-20

**Authors:** Kenta Tomihara, Ana Llopart, Daisuke Yamamoto

**Affiliations:** Advanced ICT Research Institute, National Institute of Information and Communications Technology, Kobe, Hyogo 651-2492, Japan; Interdisciplinary Graduate Program in Genetics, University of Iowa, Iowa City, IA 52242, USA; Department of Biology, University of Iowa, Iowa City, IA 52242, USA; Advanced ICT Research Institute, National Institute of Information and Communications Technology, Kobe, Hyogo 651-2492, Japan

**Keywords:** *Drosophila madeirensis*, genome assembly, HiFi, inversion breakpoint

## Abstract

*Drosophila subobscura* is distributed across Europe, the Near East, and the Americas, while its sister species, *Drosophila madeirensis*, is endemic to the island of Madeira in the Atlantic Ocean. *D. subobscura* is known for its strict light-dependence in mating and its unique courtship displays, including nuptial gift-giving. *D. subobscura* has also attracted the interest of researchers because of its abundant variations in chromosomal polymorphisms correlated to the latitude and season, which have been used as a tool to track global climate warming. Although *D. madeirensis* can be an important resource for understanding the evolutionary underpinning of these genetic characteristics of *D. subobscura*, little work has been done on the biology of this species. Here, we used a HiFi long-read sequencing data set to produce a de novo genome assembly for *D. madeirensis*. This assembly comprises a total of 111 contigs spanning 135.5 Mb and has an N50 of 24.2 Mb and a BUSCO completeness score of 98.6%. Each of the 6 chromosomes of *D. madeirensis* consisted of a single contig except for some centromeric regions. Breakpoints of the chromosomal inversions between *D. subobscura* and *D. madeirensis* were characterized using this genome assembly, updating some of the previously identified locations.

## Introduction

The genus *Drosophila* contains over 1,600 species (O’Grady and DeSalle 2018), which are highly divergent in morphology and behavior (Kopp *et al.* 2000; Prud’homme *et al.* 2006; Tanaka *et al.* 2009; Baker *et al.* 2024). Among these, *Drosophila melanogaster* is one of the best-studied organisms in the animal kingdom: a large collection of mutants and genetically modified fly stocks as well as sophisticated genetic techniques have made it an unparalleled model for studies in all biological disciplines. Because *D. melanogaster* can be genetically modified in numerous ways, the functions of a large number of genes have already been unveiled using this species. Thus, comparative approaches with members of the genus *Drosophila* will benefit enormously from the knowledge accumulated by the studies in *D. melanogaster*. *Drosophila subobscura* offers a good starting point for such comparative approaches because both classic genetics at the chromosomal level and modern molecular genetics, including genome sequence data, are publicly available for this species as potential substrates for addressing specific scientific questions.

*D. subobscura* was originally found in Europe (Buzzati-Traverso and Scossiroli 1955) and was more recently introduced to the Americas (Prevosti *et al.* 1988; Ayala *et al.* 1989). *D. subobscura* is known for a few unique displays in courtship behavior, such as nuptial gift-giving (Steele 1986a, 1986b; Tanaka *et al.* 2017), an absolute light requirement for mating (Philip *et al.* 1944; Wallace and Dobzhansky 1946; Keesey *et al.* 2020), an absence of courtship songs (Ewing and Bennet-Clark 1968), and monoandry (Maynard Smith 1956; Fisher *et al.* 2013). The karyotype of *D. subobscura* consists of 5 large chromosomes named O, U, J, A, and E, and a small dot chromosome, which are also termed Muller elements E, B, D, A, C, and F, respectively (Karageorgiou *et al.* 2019). Many variations in the sequence arrangement have been found across all 5 large chromosomes (reviewed in Sperlich and Pfriem 1986). Inversion polymorphisms show adaptive variation patterns across latitudes (Ayala *et al.* 1989) and seasons (Rodríguez-Trelles *et al.* 1996; Rodríguez-Trelles 2003). Thus, chromosomal inversion polymorphisms of *D. subobscura* have been used to track global climate warming (Rodríguez-Trelles and Rodríguez 1998; Balanyá *et al.* 2006). Recent long-read–based whole-genome sequencing helped unravel the link between inversions and seasonal adaptation in *D. subobscura* (Karageorgiou *et al.* 2020).

**Fig. 1.**
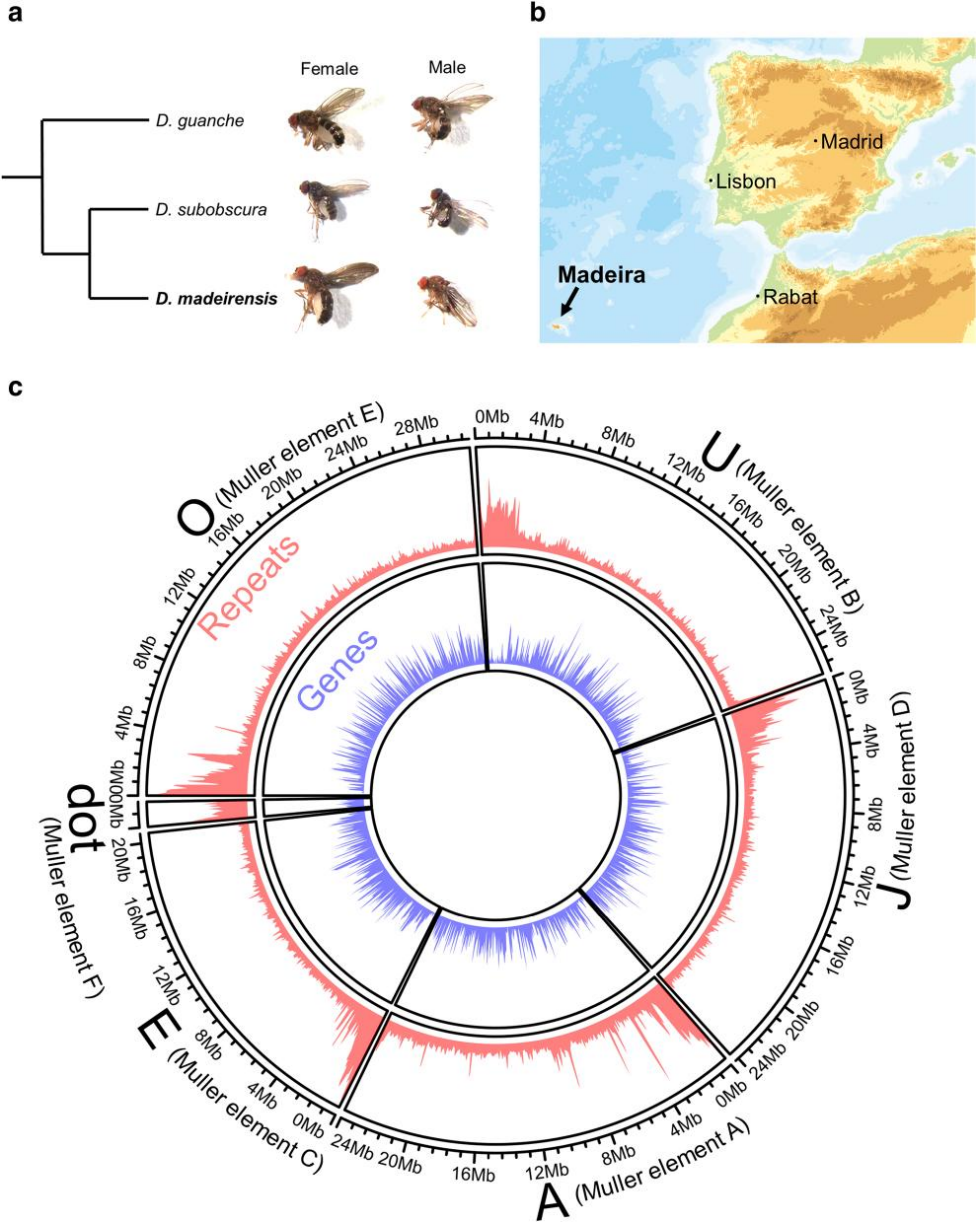
Overview of *D. madeirensis* and its genome. a) A phylogenetic tree and pictures of fly samples of the species belonging to the *D. subobscura* subgroup. b) Distribution of *D. madeirensis*. *D. madeirensis* is endemic to the island of Madeira in the Atlantic Ocean. The map was modified from the digital map published by the Geospatial Information Authority of Japan. c) Representation of the distribution of genes and repeat sequences on each *D. madeirensis* assembled chromosome. Chromosomes are ordered by their size. The proportion of elements per each 100-kb nonoverlapping window is plotted as a histogram. The *y*-axis range is set to 0–1. The circular genome map was produced using the circlize R package (Gu *et al.* 2014).

The *subobscura* subgroup consists of three species, namely, *D. subobscura*, *Drosophila madeirensis*, and *Drosophila guanche* (Fig. 1a; Barrio *et al.* 1994; Barrio and Ayala 1997; Gao *et al.* 2007). These 3 species are morphologically very similar, although the adults of *D. madeirensis* and *D. guanche* are larger in size and lighter in color than those of *D. subobscura* (Fig. 1a). Additionally, the wings of *D. madeirensis* are clouded toward tip and along the costal vein, which is different from those of the other 2 species (Monclús 1984). Among them, *D. subobscura* and *D. madeirensis* are particularly closely related and are believed to have diverged allopatrically about 0.6–1.0 million years ago (Ramos-Onsins *et al.* 1998; Herrig *et al.* 2014). *D. madeirensis* is endemic to Madeira, an island in the Atlantic Ocean located ∼580 km west of Morocco (Fig. 1b; Monclús 1984). Under laboratory conditions, *D. subobscura* and *D. madeirensis* can mate, and some of their F_1_ hybrids are fertile (summarized in Supplementary Table 1). In nature, limited gene flow was detected between *D. subobscura* on Madeira and *D. madeirensis* (Herrig *et al.* 2014). The hybrid offspring displays a range of anomalies, including dwarf testis, immotile sperm, reduced viability, extra sex combs, and aberrant head and abdomen shape (Papaceit *et al.* 1991; Khadem and Krimbas 1993). No previous work has compared the behaviors of these species, although a unique courtship ritual, nuptial gift-giving, is known in *D. subobscura*. Obviously, *D. madeirensis* will provide an ideal platform for studying the molecular, cellular, and neural bases of species barrier and speciation processes when its genome sequence becomes available. However, while a substantial body of knowledge has been accumulated on the biology of *D. subobscura*, work on *D. madeirensis* has been sparse. In the present study, a highly complete and contiguous de novo genome assembly for *D. madeirensis* was constructed using HiFi long-read sequencing, which will serve as an important resource for future studies in all biology disciplines in this species subgroup.

## Materials and methods

### Insects

The *D. madeirensis* strain RF1 was reared at 18°C on standard cornmeal–yeast–agar medium and kept in an incubator on a 12-h light/dark cycle. This strain was established from a single mated female (i.e. isofemale line) collected in Ribeiro Frio, Madeira (Herrig *et al.* 2014).

### Genome extraction and sequencing

Ten adult females were collected in 1 tube, and genomic DNA was extracted using a Genomic-tip 20/G kit (QIAGEN, Hilden, Germany) according to the manufacturer's protocol. A library for HiFi sequencing was prepared using a SMRTbell Prep Kit 3.0 (Pacific Biosciences, San Diego, CA), and sequencing was performed with a PacBio Sequel II system (Pacific Biosciences). Library preparation and HiFi genome sequencing were performed by Takara Bio (Shiga, Japan). A library for short-read sequencing was prepared using a NEBNext Ultra II DNA Library Prep kit (New England Biolabs, Beverly, MA) and sequenced using a NovaSeq 6000 (Illumina, San Diego, CA) with 150 bp paired-end reads. Library preparation and genome sequencing for short-read sequencing were performed by Novogene (Beijing, China).

### Genome size estimation

Low-quality reads and adapter sequences were removed from raw short reads using Trim Galore! (https://www.bioinformatics.babraham.ac.uk/projects/trim_galore/; Quality Phred score cutoff, 20; maximum trimming error rate, 0.1; minimum required adapter overlap, 1 bp; minimum required sequence length for both reads before a sequence pair is removed, 20 bp). The optimal *k*-mer length for this data set was estimated by KmerGenie (Chikhi and Medvedev 2014), and then the genome size of *D. madeirensis* was estimated using this *k*-mer value (*k*-mer = 111).

### Genome assembly

HiFi reads from PacBio Sequel II were assembled with Hifiasm (Cheng *et al.* 2021), HiCanu (Nurk *et al.* 2020), PacBio's Improved Phased (IPA; https://github.com/PacificBiosciences/pbipa), Flye (Kolmogorov *et al.* 2019), and NextDenovo (Hu *et al.* 2024). The options used in the assembler tools are listed in Supplementary Table 2. Assembly statistics and completeness were calculated by QUAST-LG (Mikheenko *et al.* 2018) and BUSCO (Waterhouse *et al.* 2018), respectively. In BUSCO, consensus sequences were searched against the diptera_odb10 dataset. Because NextDenovo assembled most of the nuclear genome and the genome size matched that estimated by the *k*-mer method, we used this assembly for further analyses (see Results and discussion).

The custom repeat library for the genome sequence assembled by NextDenovo was generated by RepeatModeler (Flynn *et al.* 2020). Based on this library, repetitive sequences were masked from the assembled genome by RepeatMasker (http://www.repeatmasker.org). For each masked contig, BLASTn was performed against the NCBI nt database, and the sequence with the highest homology was obtained (options: -task megablast -evalue 1 × 10^−10^ -soft_masking true). Contigs that were assigned to the sequences derived from bacterial species were removed from the genome assembly. To calculate coverage, HiFi and Illumina short reads were mapped to this assembly using minimap2 (Li 2018) with the map-hifi and sr options, respectively.

To compare the quality of genome assemblies in the *D. subobscura* subgroup, reference sequences and genomic short reads of *D. subobscura* (Bracewell *et al.* 2019) and *D. guanche* (Puerma *et al.* 2018) were obtained from public databases (*D. subobscura* reference, GCF_008121235.1; *D. subobscura* short reads, SRR9967659; *D. guanche* reference, dgua6.chromosomes.v2.fasta in https://denovo.cnag.cat/dguanche; *D. guanche* short reads, ERR2036500; all accession numbers are for NCBI). QUAST-LG and BUSCO were performed as above. Merqury was also performed to calculate consensus quality values (Rhie *et al.* 2020).

### RNA extraction, sequencing, and gene annotations

Total RNA was extracted from embryos, larvae, pupae, and adults (Supplementary Table 3) using TRIzol reagent (Thermo Fisher Scientific, Waltham, MA) according to the manufacturer's protocol. Poly-A selected libraries were prepared with a NEBNext Ultra II Directional RNA Library Prep Kit (New England Biolabs) and sequenced using the NovaSeq 6000 (Illumina) with 150 bp paired-end reads. Library preparation and sequencing were performed by Novogene.

Low-quality reads and adapter sequences were removed from raw reads using Trim Galore! with the same options as for genomic short reads. Gene models were predicted by BRAKER3 (Gabriel *et al.* 2023) using a repeat-masked genome assembly, trimmed RNA-seq reads, and a database of protein families across arthropods obtained from OrthoDB (Kuznetsov *et al.* 2023). The gene models were annotated using a DIAMOND BLASTp search (Buchfink *et al.* 2021) against the NCBI RefSeq database.

### Alignment of *D. madeirensis* and *D. subobscura* genome sequences

The *D. madeirensis* genome was aligned to the *D. subobscura* reference genome (Bracewell *et al.* 2019) by minimap2 (Li 2018) with the asm5 option. Harr plots were drawn by pafr (https://github.com/dwinter/pafr) and a custom R script.

## Results and discussion

### Genome assembly

With the HiFi data set, we compared the performance of 5 assemblers: Hifiasm (Cheng *et al.* 2021), HiCanu (Nurk *et al.* 2020), IPA (https://github.com/PacificBiosciences/pbipa), Flye (Kolmogorov *et al.* 2019), and NextDenovo (Hu *et al.* 2024) (Table 1). There were large differences in contig continuity. The largest contigs in Hifiasm and HiCanu were assembled to 43.2 Mb, whereas those of NextDenovo, IPA, and Flye were assembled to 30.8, 30.6, and 19.2 Mb, respectively. NextDenovo and HiCanu showed the two highest N50 values (24.2 and 21.2 Mb, respectively) among the assemblers (3.3–16.1 Mb). Overall, Hifiasm, HiCanu, and NextDenovo performed better than the rest of the assemblers in terms of contig continuity. The total contig lengths also varied substantially according to the assembler (Table 1; 146.3–328.2 Mb). Using the *k*-mer method, we evaluated the genome size of *D. madeirensis* to be 140.6 Mb. A previous work estimated the genome size of *D. subobscura* to be about 137 Mb by *k*-mer and 148 Mb by flow cytometry (Karageorgiou *et al.* 2019), in line with our estimation for the *D. madeirensis* genome. Taking these results together, we consider that the size of the total contig lengths was correctly estimated by NextDenovo but overestimated by the other 4 assemblers. Therefore, we conclude that the genome sequence assembled by NextDenovo would be the most reliable in regard to both the contig continuity and genome size.

**Table 1.** Statistics of genomes assembled by 5 different assemblers.

Assembler	QUAST-LG					BUSCO (3,285 groups searched)			
	Number of contigs	Total length (bp)	Largest contig (bp)	N50 (bp)	L50	Complete and single-copy	Complete and duplicated	Fragmented	Missing
Hifiasm	226	292,387,389	43,242,118	21,175,663	5	3,203	54	17	11
HiCanu	1,478	328,219,170	43,238,710	16,072,932	8	3,081	176	17	11
IPA	110	174,598,151	30,560,687	9,811,834	6	3,240	17	17	11
Flye	747	273,503,793	19,173,260	3,263,947	16	3,172	85	17	11
NextDenovo	146	146,286,141	30,799,563	24,201,710	3	3,241	18	15	11
Final assembly (contaminants removed)	111	135,508,623	30,799,563	24,201,710	3	3,240	18	16	11

To identify possible contaminants in the contigs obtained with NextDenovo, we performed BLASTn searches against the NCBI nt genome database. Out of 146 contigs, 24 were assigned to *Gluconobacter* (5.5 Mb in total), 9 were assigned to *Serratia* (5.1 Mb), 1 was assigned to *Klebsiella* (85 kb), and 1 was assigned to *Komagataeibacter* (45 kb) bacterial species. All these bacteria are inhabitants of the *Drosophila* gut (Staubach *et al.* 2013; Chaston *et al.* 2016; Ramírez-Camejo *et al.* 2017; Gao *et al.* 2023). We analyzed the sequence after removing these contigs derived from bacteria. The average coverage of HiFi reads across this assembly was 89.67×. This assembly comprised a total of 111 contigs spanning 135.5 Mb and had an N50 of 24.2 Mb (Table 1). Of the 3,285 dipteran BUSCOs, 3,240 (98.6%) were found to be complete and single-copy (Table 1). All 6 chromosomes of *D. madeirensis* were assembled into a single contig (Fig. 1), except for some centromeric regions (see below). The quality of this assembly is comparable to that of two other *D. subobscura* subgroup species (Supplementary Table 4).

### Gene annotation

Repeat sequences were predicted using RepeatMasker, which covered 19.5% of the whole genome (Fig. 1c). Using RNA-seq data and a protein data set across arthropods obtained from OrthoDB (Kuznetsov *et al.* 2023), BRAKER3 (Gabriel *et al.* 2023) annotated 13,789 protein-coding genes in the *D. madeirensis* genome, which would potentially produce 17,455 unique proteins (Fig. 1c).

Centromeres in many multicellular eukaryotes consist of highly repetitive DNA sequences (Melters *et al.* 2013). In *D. subobscura*, all 5 large chromosomes are telocentric (i.e. the centromeres are at the end of chromosomes; Buzzati-Traverso and Scossiroli 1955; Bracewell *et al.* 2019). We found that repetitive DNA sequences in the *D. madeirensis* genome are concentrated at the end of chromosomes where gene density is also lower (Fig. 1c). This pattern is similar to that of *D. subobscura* (Bracewell *et al.* 2019), suggesting that *D. madeirensis* also has telocentric chromosomes. However, it is important to note that analyses of centromeric regions are technically challenging and may have not been fully assembled in this study: the presence of repetitive sequences hampered reliable assembly of small contigs into 6 chromosomes of *D. madeirensis*. Illumina short reads and PacBio HiFi reads mapped uniformly across most genomic regions, except for some centromeric regions, where significant fluctuations in the coverage were found, indicative of the reduced quality of assembly in these regions (Supplementary Fig. 1).

### Chromosomal structures of *D. madeirensis* and *D. subobscura*

**Fig. 2.**
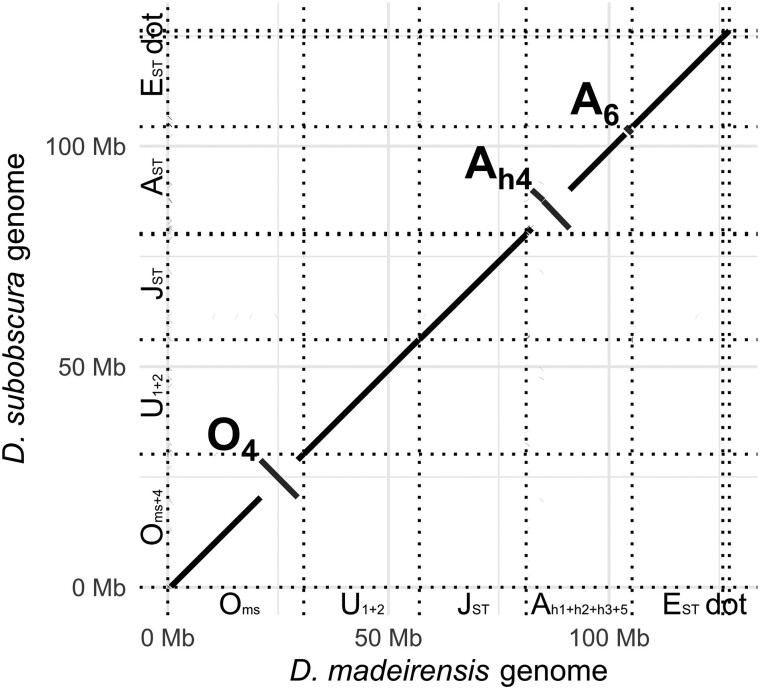
Harr plot made with minimap2 (Li 2018) whole-genome alignment between *D. madeirensis* and *D. subobscura* genome sequences. Sequences aligned in forward and reverse orientations are represented by upward-sloping and downward-sloping lines, respectively.

Figure 2 shows the genome alignment of *D. madeirensis* and the *D. subobscura* reference strain (14011-0131.10; Bracewell *et al.* 2019). All 6 *D. subobscura* chromosomes were uniquely aligned with the *D. madeirensis* chromosomes, demonstrating the contiguity and completeness of both assemblies. In previous studies, the chromosomal structure of *D. madeirensis* was referred to as O_ms_, U_1 + 2_, J_ST_, A_h1 + h2 + h3 + 5_, and E_ST_, while that of the *D. subobscura* 14011-0131.10 strain was called O_ms + 4_, U_1 + 2_, J_ST_, A_ST_, and E_ST_ (Karageorgiou *et al.* 2019, 2020). There are no obvious structural rearrangements of chromosomes U, J, and E between the two species, so that the same names are used to describe them. In contrast, there are large inversions on chromosomes A and O and a small additional inversion at the end of chromosome A of *D. madeirensis*, consistent with previous polytene chromosome analyses of the hybrids between *D. madeirensis* and *D. subobscura* (Krimbas and Loukas 1984; Papaceit and Prevosti 1989).

**Fig. 3.**
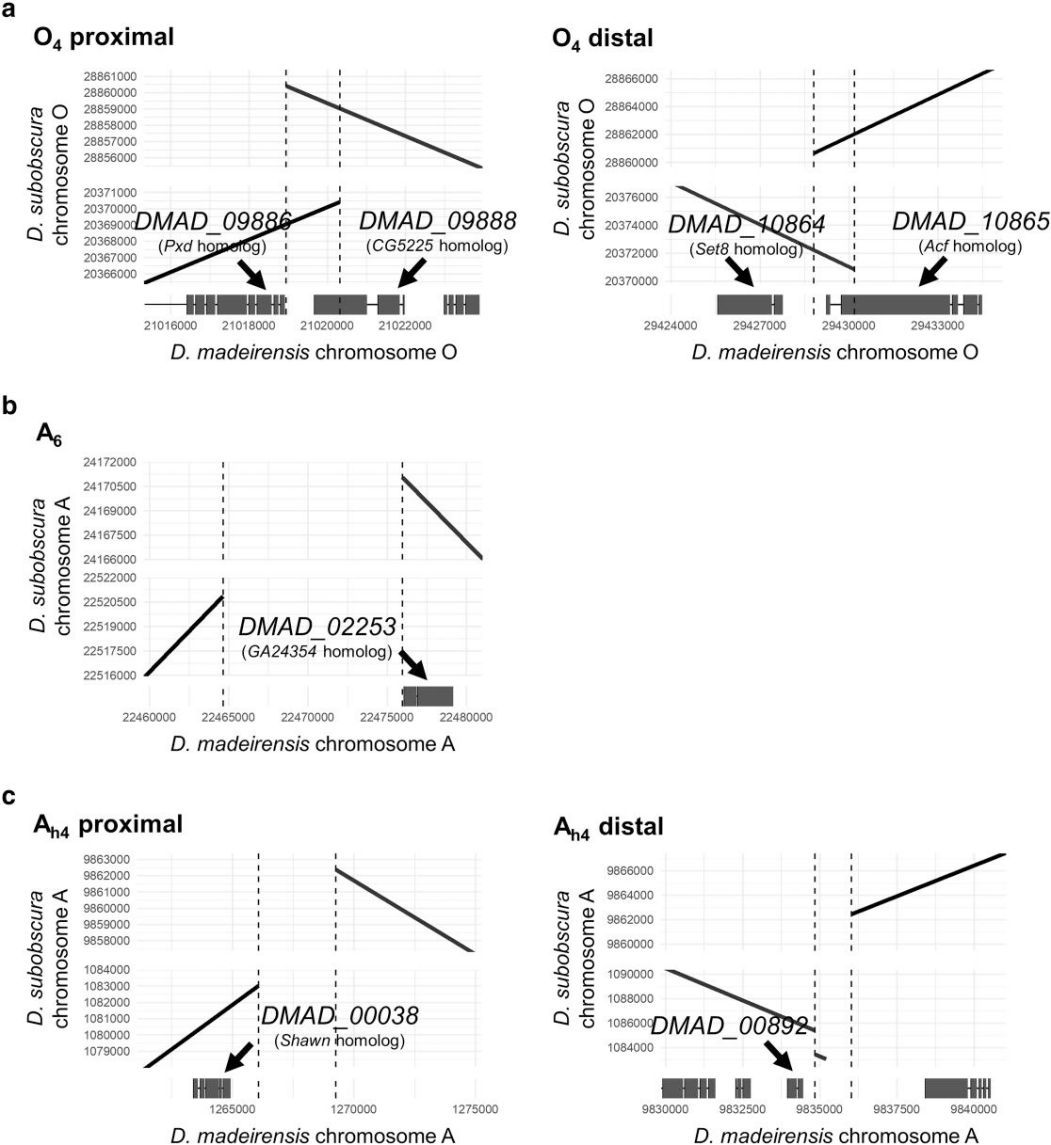
Detailed characterization of inversion breakpoints. Harr plots of *D. madeirensis* and *D. subobscura* genome sequences at the O_4_ a), A_6_ b), and A_h4_ c) breakpoints are shown. Sequences aligned in forward and reverse orientations are represented by upward-sloping and downward-sloping lines, respectively. The dotted lines indicate breakpoints of the inversions. Each filled rectangle represents a coding region of the *D. madeirensis* gene model.

Breakpoints of inversion O_4_ found in the *D. subobscura* O_ms+4_ chromosome were previously characterized using different *D. subobscura* strains: the proximal O_4_ breakpoint contains two genes, i.e. *Peroxidase* (*Pxd*) and *CG5225*, while the distal O_4_ inversion breakpoint contains another two genes, i.e. *SET domain containing 8* (*Set8*) and *ATP-dependent chromatin assembly factor large subunit* (*Acf*; Puerma *et al.* 2016). Here, the gene names are based on the *Drosophila melanogaster* ortholog. Our alignment of *D. subobscura* and *D. madeirensis* genomes confirmed that the O_4_ inversion occurred at these breakpoints (Fig. 3a; Supplementary Table 5): the inversion O_4_ involved the coding regions of *CG5225* and *Acf*. The proximal and distal inversion breakpoints of O_4_ were both duplicated, and hence, upward-sloping and downward-sloping diagonals were drawn to overlap at the breakpoints in Fig. 3a. Due to this duplication event, *D. subobscura* should have an intact copy of each of *CG5225* and *Acf* in addition to a disrupted copy of *CG5225* and *Acf*. Indeed, the genome database shows that *D. subobscura* has the full length *CG5225* and *Acf* genes (NCBI accession numbers XP_034663006 and XP_034663002). The detailed information about the structure of this inversion is available in Puerma et al. (2016).

A_h1+h2+h3+5_ and A_ST_ were predicted to have 2 inversions, namely, A_h4_ and A_6_ (Karageorgiou *et al.* 2019). Because A_6_ is a terminal inversion, it has only 1 breakpoint. By designing molecular markers on the *D. subobscura* and *D. guanche* genomes, Orengo et al. (2019) mapped the A_6_ breakpoint to an ∼20-kb-long region flanked by genes homologous to *Drosophila pseudoobscura GA22805* and *GA24354* (note that A_6_ and A_h4_ were referred to as A_f6_ and A_f1_ in their study, respectively). Our alignment of the *D. subobscura* and *D. madeirensis* genomes further narrowed the A_6_ breakpoint to a 11.3-kb region near *DMAD_02253*, a gene homologous to *GA24354* (Fig. 3b, Supplementary Table 5), which supports the result of the previous study. Orengo et al. (2019) also determined the proximal and distal breakpoints of A_h4_: an ∼44-kb region flanked by the genes homologous to *D. pseudoobscura GA14783* (corresponding to the *D. madeirensis* gene *DMAD_00872*) and *GA13678* (*DMAD_00079*), and an ∼33-kb region flanked by *GA17070* (*DMAD_00099*) and *GA15499* (*DMAD_00868*), respectively. However, our alignment of the *D. subobscura* and *D. madeirensis* genomes gave a different result. We located the proximal A_h4_ breakpoint on a 3.2-kb region near *DMAD_00038* and the distal breakpoint on a 1.2-kb region near *DMAD_00892* (Fig. 3c; Supplementary Table 5). The proximal and distal A_h4_ breakpoints determined in this study were about 521 and 87 kb away from the closest genes characterized by Orengo et al. (2019), respectively (Supplementary Fig. 2 and Table 5). We found many inversions on the A chromosome that had occurred after the separation of *D. guanche* from the clade of *D. subobscura*/*D. madeirensis* (Supplementary Fig. 3), and this may have complicated the previous mapping of the A_h4_ breakpoints, which relied on molecular markers designed solely based on the genomic information of *D. guanche* and *D. subobscura* (Orengo *et al.* 2019). These considerations highlight the importance of using closely related lineages with fewer chromosomal rearrangements in mapping chromosomal breakpoints.

The comprehensive knowledge of the *D. madeirensis* genome obtained in this study will provide a solid basis for future comparative analyses of the diverged phenotypic traits found among the three members of the *D. subobscura* species subgroup.

## Supplementary Material

jkae167_Supplementary_Data

## Data Availability

The raw sequence data have been submitted to DDBJ under accession numbers DRR528066 (genomic HiFi reads), DRR528092 (genomic short reads), and DRR528093-528099 (RNA-seq reads). The *D. madeirensis* genome assembly and gene models have been submitted to DDBJ under accession numbers AP029263-AP029373. Supplemental material available at G3 online.
