# Supplementary material for: A chromosome-level genome assembly of Drosophila madeirensis, a fruit fly species endemic to the island of Madeira
Source: G3 (Bethesda). 2024 Jul 20;14(9):jkae167. doi: 10.1093/g3journal/jkae167 (PMC11373663; doi:10.1093/g3journal/jkae167)
Supplement: jkae167_Supplementary_Data [file jkae167_supplementary_data.zip › Supplemental_Figures_G3-2024-405197.docx]

**Supplemental Figures**

**A chromosome-level genome assembly of *Drosophila madeirensis*, a fruit fly species endemic to the island of Madeira**

Kenta Tomihara^1^*, Ana Llopart^2,3^, Daisuke Yamamoto^1^*

^1^Neuro-ICT Laboratory, Advanced ICT Research Institute, National Institute of Information and Communications Technology, Kobe, Hyogo, Japan

^2^Interdisciplinary Program in Genetics, University of Iowa, Iowa City, Iowa, USA

^3^Department of Biology, University of Iowa, Iowa City, Iowa, USA


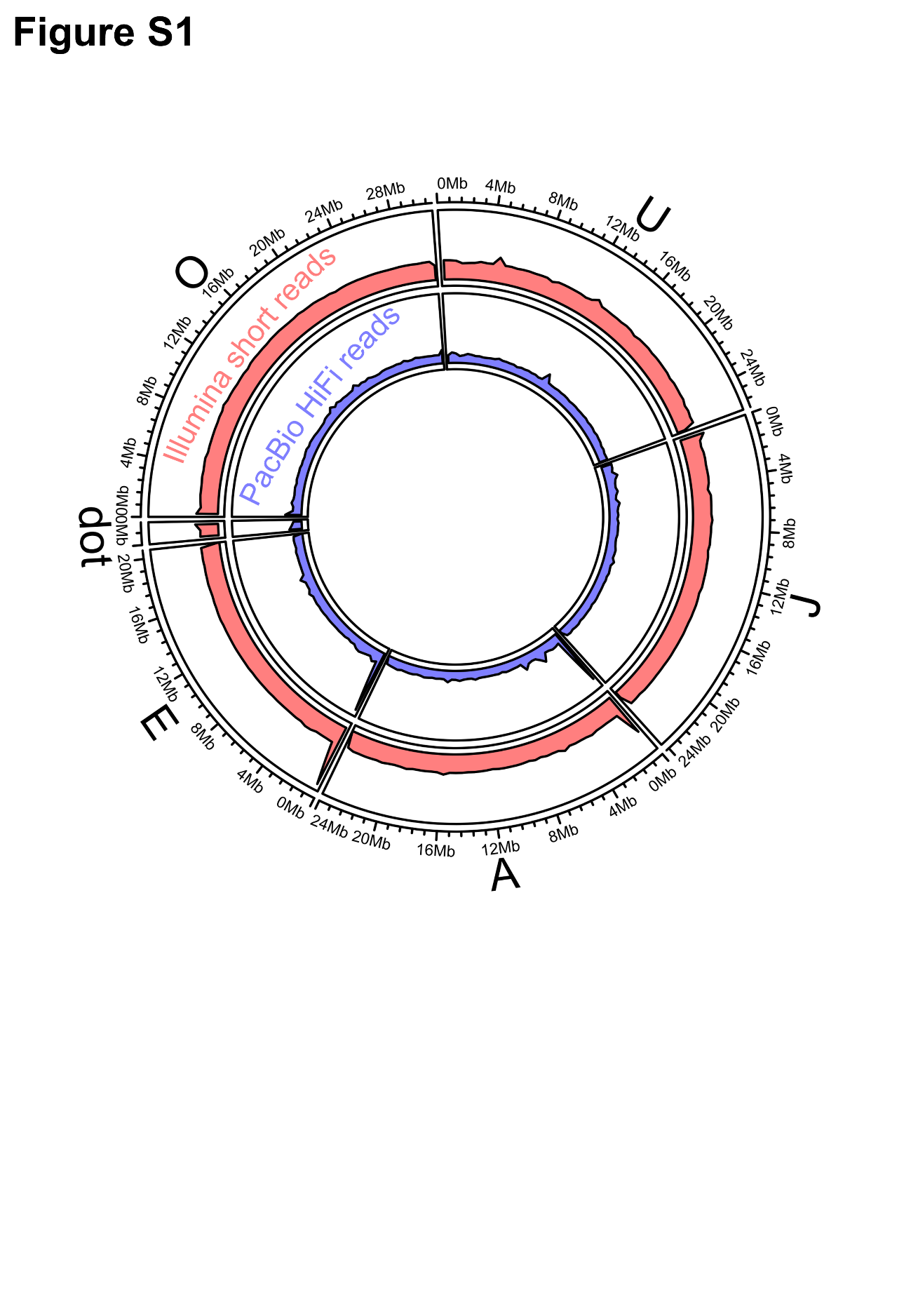
**Figure S1.** Coverage plot of Illumina short and PacBio HiFi reads. The coverage depths per each 500-kb non-overlapping window are plotted as a histogram.

**
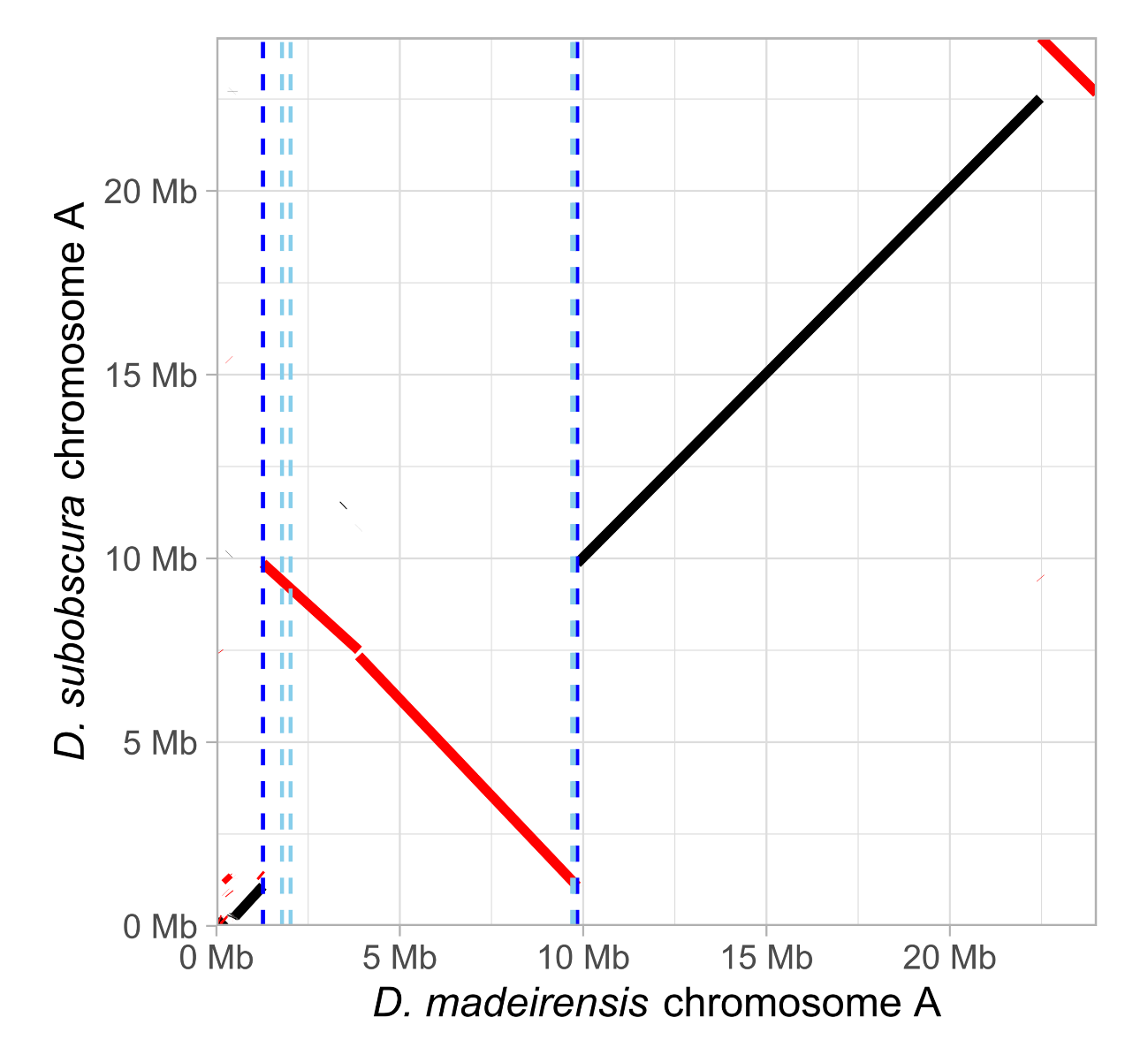
Figure S2.** Harr-plot analysis of *D. madeirensis* and *D. subobscura* chromosome A sequences. The blue and sky-blue dotted lines indicate breakpoints of the inversions characterized in this study and Orengo et al. (2019), respectively.

**
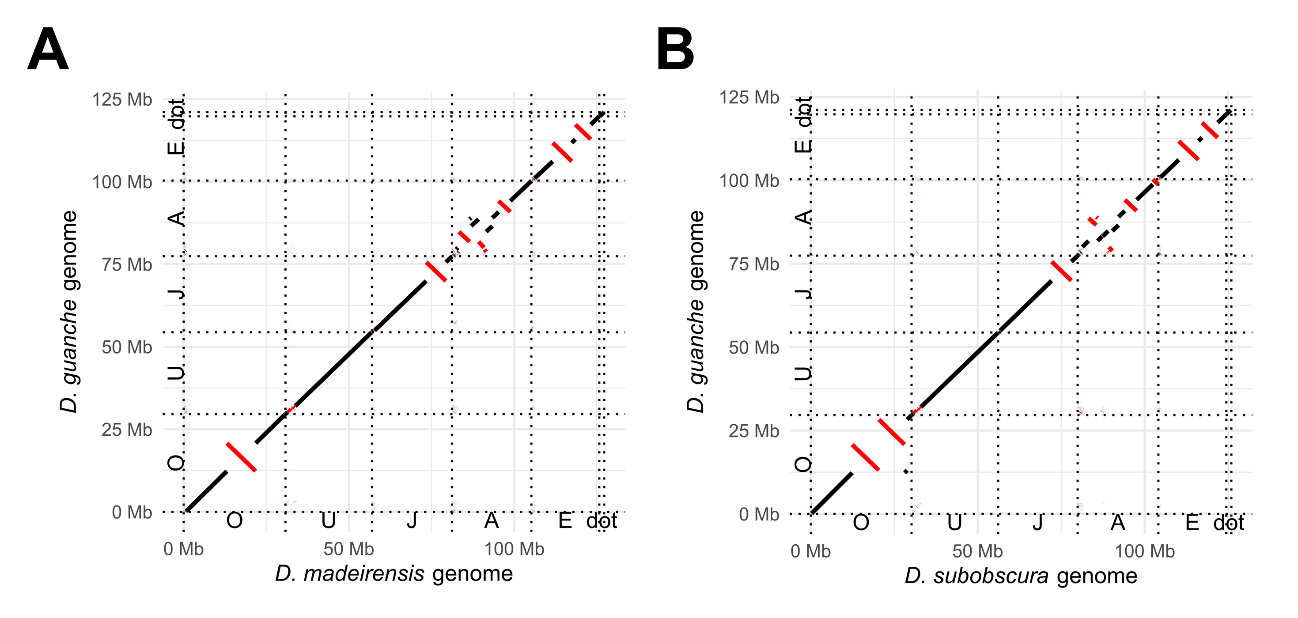
Figure S3.** Harr-plot analysis of the *D. guanche* and *D. madeirensis* (A), and *D. guanche* and *D. subobscura* (B) genome sequences. Sequences aligned in the forward and reverse orientations are represented by black and red lines, respectively.

**References**

Orengo, D. J., E. Puerma, and M. Aguadé, 2019 The molecular characterization of fixed inversions breakpoints unveils the ancestral character of the *Drosophila guanche* chromosomal arrangements. Sci Rep 9: 1706.
